# Impact of Early-Onset Sepsis on the Development and Severity of Intraventricular Hemorrhage in Premature Neonates: A Retrospective Single-Center Study

**DOI:** 10.3390/children13081004

**Published:** 2026-07-29

**Authors:** Halil Kul, Harun Demirci, Sara Erol, Avni Merter Keceli, Sima Cebecik Çakır, Mahmut Sami Çolak, Pınar Özışık

**Affiliations:** 1 Department of Neurosurgery, Ankara Bilkent City Hospital, Faculty of Medicine, Ankara Yıldırım Beyazıt University, 06800 Ankara, Turkey; harundemirci@aybu.edu.tr (H.D.); sima.cebecikcakir@saglik.gov.tr (S.C.Ç.); mahmutsami.colak@saglik.gov.tr (M.S.Ç.); pinar.ozisik@aybu.edu.tr (P.Ö.); 2 Division of Neonatology, Department of Pediatrics, Ankara Bilkent City Hospital, Faculty of Medicine, Ankara Yıldırım Beyazıt University, 06800 Ankara, Turkey; saraerol@aybu.edu.tr; 3 Department of Radiology, Ankara Bilkent City Hospital, Faculty of Medicine, Ankara Yıldırım Beyazıt University, 06800 Ankara, Turkey; avni.merter.keceliadu@edu.tr

**Keywords:** intraventricular hemorrhage (IVH), neonatal sepsis, cerebrospinal fluid (CSF) culture

## Abstract

**Highlights:**

**What are the main findings?**

• Neonatal sepsis was significantly associated with high-grade intraventricular hemorrhage (Grades III–IV) in premature infants and increased the likelihood of severe IVH by approximately fourfold.
• All neonates with positive cerebrospinal fluid cultures were in the high-grade IVH group, and significantly lower CSF glucose levels suggested concurrent central nervous system infection or inflammation.

**What are the implications of the main findings?**

• Early recognition and prompt treatment of neonatal sepsis may help reduce the risk and severity of IVH in premature neonates.
• Careful evaluation for meningitis or CNS infection should be considered in premature infants with severe IVH to prevent infection-related brain injury and adverse neurodevelopmental outcomes.

**Abstract:**

Objective: Intraventricular hemorrhage (IVH) is a major cause of morbidity and mortality in premature infants. Early onset neonatal sepsis (EOS) may increase the risk and severity of IVH through inflammation and hemodynamic instability. This study evaluated the association between sepsis and IVH severity. Methods: This retrospective, single-center study included neonates diagnosed with IVH and followed in the Neonatal Intensive Care Unit of a tertiary hospital between January 2021 and December 2022. IVH grading was based on Papile’s classification, and patients were categorized as low-grade (Grades I–II) or high-grade (Grade III IVH and periventricular hemorrhagic infarction [PVHI], previously referred to as Grade IV IVH). Clinical and laboratory data, including cerebrospinal fluid (CSF) and blood culture results, were analyzed. Logistic regression was used to identify factors associated with high-grade IVH. Results: A total of 61 neonates were included, of whom 49 (80.3%) had high-grade IVH. Sepsis was detected in 33 of 49 patients (67.3%) with high-grade IVH, representing a statistically significant difference compared with the low-grade group (*p* = 0.047). Logistic regression analysis identified sepsis as an independent predictor of high-grade IVH, increasing the likelihood approximately fourfold. All patients with positive CSF cultures were in the high-grade IVH group, and CSF glucose levels were significantly lower (*p* < 0.05), suggesting central nervous system infection or inflammation. Conclusions: Sepsis significantly increases the risk of severe (high-grade) intraventricular hemorrhage in premature infants. Early diagnosis and prompt treatment of neonatal sepsis and meningitis are crucial for preventing infection-related brain injury and improving neurodevelopmental outcomes.

## 1. Introduction

Preterm birth remains the leading cause of neonatal and under-five mortality worldwide [1]. Intraventricular hemorrhage (IVH), one of the most common complications of preterm birth, is a significant risk factor for both mortality and neurodevelopmental disability in affected infants [2]. Despite advances in neonatal intensive care and improved survival rates of premature newborns, the morbidity associated with IVH has not declined [3]. Multiple factors contribute to the etiology of IVH, with low gestational age and low birth weight being the most prominent. The risk of IVH increases as gestational age decreases, particularly in the germinal matrix, with incidence rates ranging from 15% to 25% in infants born before 32 weeks of gestation [4,5,6]. Similarly, the incidence of IVH in premature infants weighing less than 1500 g varies between 25% and 45% [3,7,8]. Among survivors, both mild (Grades I and II) and severe IVH (Grades III and IV) are associated with an elevated risk of moderate-to-severe neurodevelopmental impairment [2,3].

Recognized risk factors for IVH include neonatal sepsis and patent ductus arteriosus, particularly in premature infants [9,10]. Neonatal sepsis is defined as a clinical syndrome characterized by systemic signs of infection accompanied by bacteremia, with the causative pathogen isolated from the blood within the first 28 days of life [11,12]. Despite improvements in diagnostic and therapeutic approaches, sepsis remains a major cause of mortality and morbidity among newborns worldwide [13]. A systematic review and meta-analysis of population-based studies estimated the pooled incidence of neonatal sepsis at 22 per 1000 live births, with a mortality rate between 11% and 19% [14]. The incidence of neonatal sepsis increases with decreasing gestational age, and the rising rates of sepsis in premature infants may contribute to an increased incidence of intraventricular hemorrhage. This study aims to evaluate the relationship between the etiopathogenesis of low- and high-grade intraventricular hemorrhage and sepsis in patients diagnosed with IVH.

## 2. Materials and Methods

### 2.1. Study Design and Population

This retrospective, single-center study was conducted at the Neonatal Intensive Care Unit of Ankara Bilkent City Hospital, a tertiary referral center with 150 neonatal intensive care beds, between January 2021 and December 2022. During the study period, all neonates admitted to this unit were screened for eligibility.

**Figure 1:**
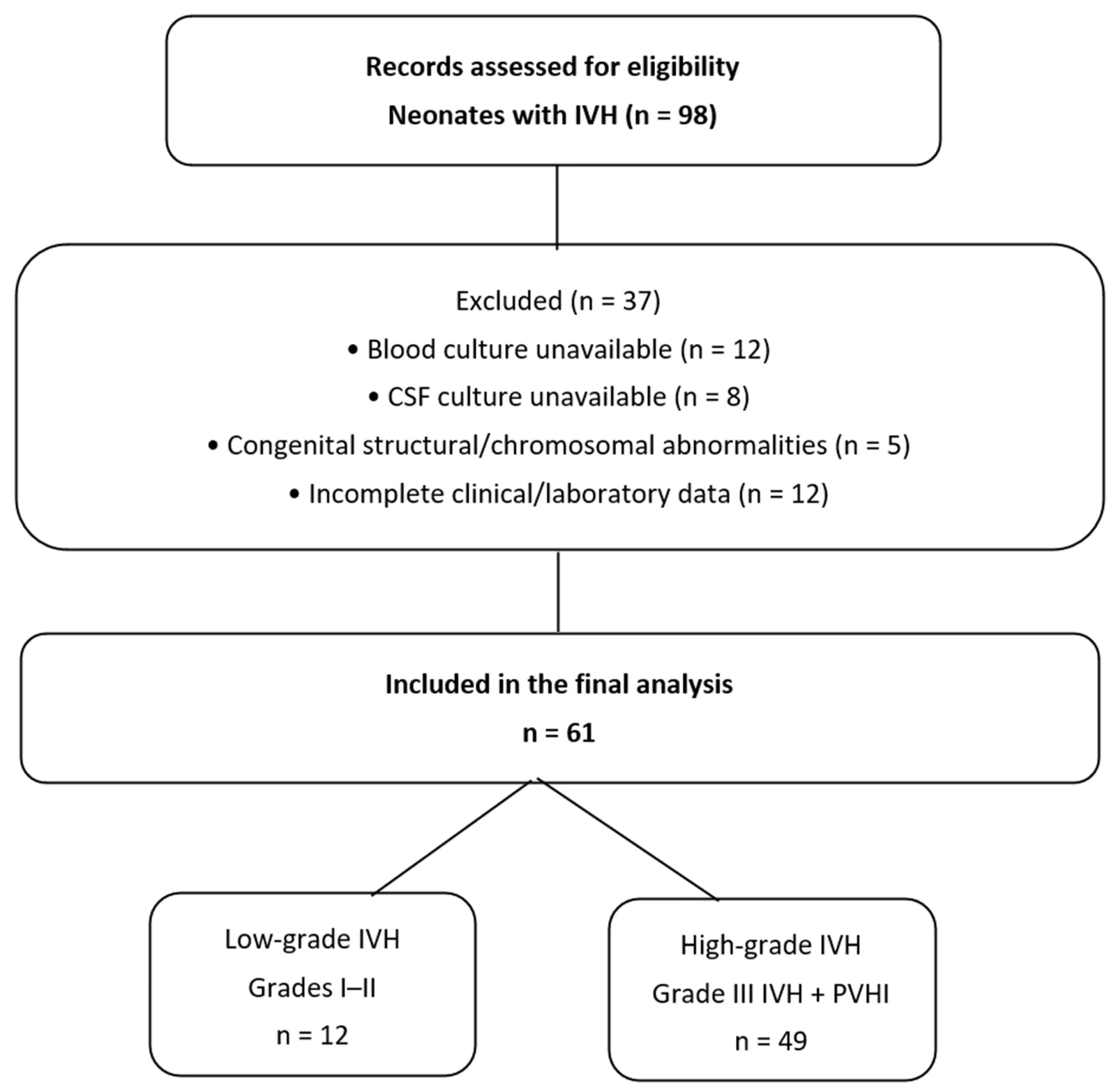
Flow diagram of patient selection and study population.

Infants who were clinically suspected of having intraventricular hemorrhage (IVH) during follow-up were evaluated using cranial imaging modalities (ultrasound and/or magnetic resonance imaging). Neonates with radiologically confirmed IVH who fulfilled the inclusion criteria were enrolled in the study (Figure 1).

### 2.2. Inclusion Criteria

Patients were included in the study if cerebrospinal fluid (CSF) samples were obtained during the diagnostic process and blood cultures were collected during follow-up. Only newborns with confirmed IVH findings on cranial ultrasound (US) or magnetic resonance imaging (MRI) were included. In addition, only patients within the neonatal period who had complete medical history and laboratory data available were considered eligible for inclusion.

### 2.3. Exclusion Criteria

Patients were excluded if they had congenital structural or chromosomal abnormalities, if IVH could not be confirmed by ultrasound or MRI, or if CSF and/or blood culture data were unavailable. The patient selection process is summarized in Figure 1.

### 2.4. Sample Collection and Laboratory Analysis

Cerebrospinal fluid samples were collected via lumbar puncture at the L3–L4 or L4–L5 interspace under sterile conditions. Povidone-iodine antiseptic solution was used for skin preparation, and a sterile drape was applied to minimize contamination. Blood samples for culture were obtained from peripheral venous vessels under sterile conditions following similar antiseptic preparation.

CSF samples were obtained during the diagnostic evaluation performed at the time of IVH diagnosis, and meningitis was assessed based on CSF culture and laboratory findings.

Sepsis was defined according to both culture-proven and clinically diagnosed criteria. For the analysis of its potential etiological association with IVH severity, only sepsis episodes diagnosed before or concurrently with the diagnosis of IVH were considered. Sepsis episodes occurring after the diagnosis of IVH were not included in the analysis. All sepsis episodes included in the analysis occurred within the first 72 h of life and were therefore classified as early-onset sepsis (EOS). The presence of sepsis was subsequently analyzed as a potential risk factor associated with IVH severity.

Culture-proven sepsis was defined as the presence of Gram-negative, Gram-positive, or fungal growth in blood or CSF cultures obtained for the diagnosis of infection.

Clinically diagnosed sepsis was defined as a systemic condition characterized by infection-related hemodynamic changes and clinical manifestations, with or without isolation of a pathogen from sterile body fluids (blood or CSF), as described by Shane et al. [10].

This definition aligns with the current neonatal sepsis literature, recognizing that culture-negative cases with clinical and laboratory evidence of infection represent a significant proportion of neonatal sepsis diagnoses.

Due to limitations inherent in retrospective data collection, information regarding maternal antenatal steroid administration and antenatal infection status could not be obtained.

### 2.5. Neuroimaging and IVH Classification

Cranial ultrasound examinations were performed according to the unit protocol within the first three days of life, repeated on day 10 and additionally when IVH was clinically suspected. MRI was reserved for selected cases requiring further evaluation of parenchymal injury, while CT was performed only in emergency situations when rapid neuroimaging was required. Cranial ultrasound and patient records from the first ten days of life were retrospectively reviewed.

The severity of IVH was classified according to the Papile Classification [15]:

• Grade I: Bleeding confined to the germinal matrix;
• Grade II: Intraventricular hemorrhage without ventricular dilatation;
• Grade III: Intraventricular hemorrhage with ventricular dilatation;
• PVHI (formerly classified as Grade IV IVH according to the Papile classification): Intraventricular and intraparenchymal hemorrhage.

For statistical analysis, patients were categorized as having low-grade IVH (Grades I–II) or high-grade IVH (Grade III IVH and periventricular hemorrhagic infarction [PVHI], formerly classified as Grade IV IVH).

### 2.6. Clinical and Laboratory Parameters

The following clinical and laboratory parameters were recorded: gestational age, birth weight, sex, comorbidities (including bronchopulmonary dysplasia and patent ductus arteriosus), blood culture results, and CSF parameters (cell count, glucose, protein, and erythrocyte count). Laboratory tests such as hemoglobin, C-reactive protein (CRP), and platelet count were also evaluated.

Newborns were categorized by gestational age as <28 weeks, 28–31 weeks, 32–35 weeks, and ≥36 weeks. Birth weight was classified as Low Birth Weight (LBW) for infants weighing <2500 g, Very Low Birth Weight (VLBW) for infants weighing <1500 g, and Extremely Low Birth Weight (ELBW) for infants weighing <1000 g.

Patients were further divided into two groups according to IVH grade—low-grade IVH (Grades I–II) and high-grade IVH (Grades III–IV)—and the presence of culture-proven or clinically diagnosed sepsis was compared between groups to assess the relationship between sepsis and IVH severity.

Approval was received from Ankara Bilkent City Hospital No 1 Clinical Research Ethics Committee for the present study with decision number 3678 on 7 June 2023.

The requirement for informed consent was waived by the Ethics Committee because of the retrospective study design and the use of anonymized patient data.

### 2.7. Statistical Analysis

Data were analyzed using IBM SPSS Statistics version 26.0 (IBM Corporation, Armonk, NY, USA). Continuous variables were tested for normality using the Kolmogorov–Smirnov test and presented as mean ± standard deviation or median (minimum–maximum), as appropriate. Categorical variables were expressed as counts and percentages. Group comparisons were performed using the independent-samples *t*-test or Mann–Whitney U test for continuous variables and the chi-square or Fisher’s exact test for categorical variables. Variables with a *p*-value < 0.1 in univariate analysis were entered into a multivariable logistic regression model to identify independent factors associated with high-grade IVH. The results were expressed as odds ratios (OR) with 95% confidence intervals (CI). Model fit was assessed using the Hosmer–Lemeshow test, Omnibus test, and Nagelkerke R^2^ statistic. A two-sided *p*-value < 0.05 was considered statistically significant.

## 3. Results

A total of 61 neonates, including 24 females, were included in the study. High-grade IVH was identified in 49 infants (80.3%). The mean birth weight was 1405 g (range: 970–1888 g), and the mean gestational age was 30 weeks. Demographic characteristics are summarized in Table 1.

A statistically significant difference in sex distribution was observed according to IVH severity (*p* = 0.047). While female sex predominated in the low-grade IVH group, male infants were more frequent in the high-grade IVH group. Among high-grade IVH patients, 41 (83.7%) had a gestational age ≤32 weeks, and 42 (85.7%) had a birth weight ≤2500 g. In contrast, in the low-grade group, eight infants (66.7%) had a gestational age between 28 and <32 weeks, and five infants (41.7%) had a birth weight between 1500 and <2500 g. Although the differences did not reach statistical significance, lower gestational age and lower birth weight were more common among infants with high-grade IVH.

In the present study, sepsis was detected in 33 of 49 (67.3%) patients with high-grade IVH, representing a statistically significant difference compared with the low-grade group (*p* = 0.047) (Table 2). In the logistic regression analysis, sepsis was identified as a significant independent factor associated with high-grade IVH (Table 3).

**Figure 2:**
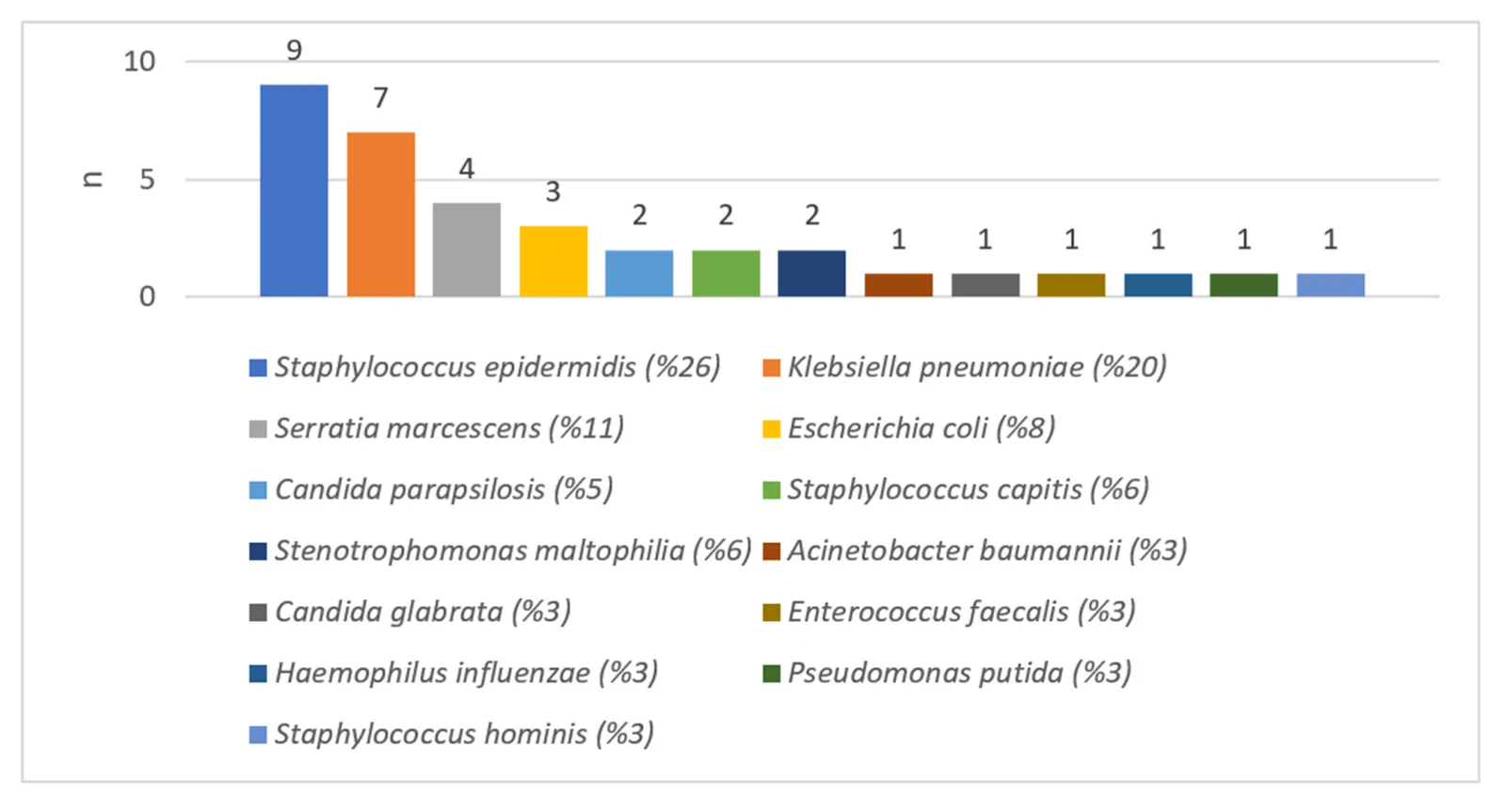
Distribution of microorganisms isolated from blood cultures in neonates with intraventricular hemorrhage.

The distribution of isolated bacterial and fungal pathogens is presented in Figure 2. Among the isolated pathogens, *Staphylococcus epidermidis* was the most frequently identified microorganism (*n* = 9, 26%), followed by *Klebsiella pneumoniae* (*n* = 7, 20%), *Serratia marcescens* (*n* = 4, 11%), *Escherichia coli* (*n* = 3, 8%), *Candida parapsilosis* (*n* = 2, 5%), and *Staphylococcus capitis* (*n* = 2, 5%). Less frequently isolated pathogens included *Stenotrophomonas maltophilia* (*n* = 2, 5%), *Acinetobacter baumannii* (*n* = 1, 3%), *Candida glabrata* (*n* = 1, 3%), *Enterococcus faecalis* (*n* = 1, 3%), *Haemophilus influenzae* (*n* = 1, 3%), *Pseudomonas putida* (*n* = 1, 3%), and *Staphylococcus hominis* (*n* = 1, 3%). (Figure 2) Among the six patients with culture-proven meningitis, cerebrospinal fluid cultures yielded *Candida glabrata* (*n* = 1), *Escherichia coli* (*n* = 1), *Acinetobacter baumannii* (*n* = 1), *Klebsiella pneumoniae* (*n* = 2), and *Staphylococcus aureus* (*n* = 1).

Evaluation of blood culture results revealed that 27 of 49 (55.1%) patients with high-grade IVH had a microbiologically confirmed pathogen. During follow-up, 13 patients died, corresponding to an overall mortality rate of 21.3%, while 10 of 49 (20.4%) deaths occurred in the high-grade IVH group. No statistically significant difference in mortality was observed between low- and high-grade IVH groups. Laboratory parameters, including hemoglobin, INR and CRP, were also evaluated; however, no statistically significant differences were found between IVH severity grades (Table 2).

## 4. Discussion

Based on the evaluation performed in our neonatal cohort, the presence of sepsis was found to be significantly associated with higher grades of intraventricular hemorrhage (IVH). According to the results of logistic regression analysis, the presence of sepsis increased the likelihood of developing high-grade IVH by approximately fourfold. In addition, lower CSF glucose levels were observed among infants with high-grade IVH, which likely reflects central nervous system inflammation secondary to infection.

Previous studies have consistently demonstrated that lower gestational age and lower birth weight are important risk factors for the development of IVH [9,16]. In accordance with these findings, our study revealed that infants with high-grade IVH had lower gestational ages and birth weights compared with those with low-grade IVH. Furthermore, in the multivariable analysis, neonates born at ≥32 to <36 weeks of gestation had significantly lower odds of high-grade IVH compared with those born before 28 weeks (OR = 0.029, 95% CI = 0.001–0.676, *p* = 0.027), supporting the protective effect of advancing gestational age against severe IVH. Although the difference in gestational age distribution between the low-grade and high-grade IVH groups did not reach statistical significance, both groups showed a predominance of neonates born at ≤32 weeks of gestation, indicating a general predisposition of extremely premature infants to IVH. Although IVH is predominantly observed in very preterm infants, it may also occur in late preterm and near-term neonates in the presence of additional risk factors. In our cohort, neonates with a gestational age of ≥36 weeks had accompanying conditions such as sepsis and cardiac comorbidities, which may have contributed to the development of IVH through alterations in cerebral blood flow and hemodynamic instability.

Furthermore, a male predominance was observed among infants with high-grade IVH in our study. Similarly, Dani et al. reported that, among premature infants, female sex was more common in low-grade IVH, whereas lower gestational age was associated with high-grade IVH [17]. In another study by Smolkova et al. investigating preterm infants, high-grade IVH was also found to be more frequent in male neonates and significantly associated with lower gestational age [18]. Consistent with the literature, our findings suggest that decreasing gestational age and birth weight not only predispose to the occurrence of IVH but also correlate with the severity of hemorrhage, contributing to the development of high-grade IVH.

Despite improved intensive care conditions and modern treatment options, neonatal sepsis continues to be a global problem [19]. Prematurity, low birth weight, chorioamnionitis, premature prolonged rupture of membranes, resuscitation, low APGAR score, inability to breastfeed, and long hospital stays are among the risk factors [20]. In studies investigating the risk factors for intraventricular hemorrhage (IVH) in premature infants, the presence of sepsis has been identified as an important risk factor and shown to increase susceptibility to IVH [10,17,21,22]. The association between sepsis and IVH is likely multifactorial. The immature germinal matrix vasculature of preterm infants is highly susceptible to injury because of structural fragility and impaired cerebral autoregulation. Systemic inflammation, hemodynamic instability, fluctuations in cerebral blood flow, and hypotension occurring during sepsis may further increase the vulnerability of these vessels and contribute to the development or progression of IVH. In addition, early-onset sepsis has been reported among the clinical factors associated with IVH in preterm infants [10,22,23,24,25]. These mechanisms may explain the observed association between sepsis and severe IVH in our cohort. In our study, in addition to the existing literature, the presence of sepsis was found to be statistically significantly associated with the occurrence of high-grade intraventricular hemorrhage (IVH). Furthermore, according to the analysis results, the presence of sepsis increased the likelihood of developing high-grade IVH by approximately fourfold. In the study conducted by Bedetti et al., which evaluated the long-term neurological effects of sepsis in preterm infants, both sepsis and IVH were identified as factors associated with severe functional disability, and this association remained statistically significant in multivariate analysis (odds ratios: 3.68 and 4.7, respectively) [26]. In conclusion, the early diagnosis and appropriate treatment of sepsis are crucial for limiting infection-related brain injury.

In our study, all six patients with positive cerebrospinal fluid cultures were in the high-grade IVH group. In addition, CSF biochemical analysis revealed that glucose levels were significantly lower in the high-grade IVH group, indicating a possible association with central nervous system infection or inflammation. In a study conducted by Zhou et al. in Canada investigating the relationship between meningitis and bloodstream infection and IVH, the incidence of grade 1 and 2 IVH in infants diagnosed with meningitis was found to be similar to that in those diagnosed with bloodstream infection. However, the incidence of grade 3 and above IVH was found to be 27.8% higher in those diagnosed with meningitis [27]. Our findings indicate that the decrease in CSF glucose levels observed in the high-grade IVH group supports the presence of central nervous system inflammation, suggesting a possible role of meningitis in etiology. This finding also implies that infectious complications in premature infants may adversely affect neurofunctional development. Although the number of patients with culture-proven meningitis was limited, meningitis may have influenced CSF biochemical findings and should be considered a potential confounding factor when interpreting the association between infection and IVH severity.

The limitations of this study include its single-center and retrospective design, which may restrict the generalizability of the findings. Additionally, data on antenatal management, including treatments administered during pregnancy and the presence of maternal infections, were not available. Furthermore, comprehensive prenatal and perinatal variables, including antenatal steroid exposure, maternal infection status, chorioamnionitis, premature rupture of membranes, and other obstetric factors that may influence the occurrence and severity of IVH, could not be reliably obtained for all patients. Consequently, these variables could not be included in the multivariable analysis. The requirement for inclusion of patients with both CSF and blood culture further reduced the sample size. Despite these limitations, the study provides valuable insight into the relationship between sepsis and intraventricular hemorrhage in premature infants. Additional inflammatory biomarkers, including procalcitonin, interleukins, and presepsin, were not routinely available and therefore could not be included in the analysis. Another limitation of this study is the lack of systematic long-term follow-up data. Consequently, neurodevelopmental and neuromotor outcomes, including hearing impairment, epilepsy, cerebral palsy, and developmental delay, could not be evaluated. Future prospective, multicenter studies with larger cohorts are needed to better elucidate the etiopathogenesis and strengthen the evidence base for clinical interventions.

## 5. Conclusions

In this study, the presence of sepsis was found to be significantly associated with the development of high-grade intraventricular hemorrhage in premature infants. Furthermore, the finding that all infants with positive cerebrospinal fluid cultures and meningitis diagnoses belonged to the high-grade IVH group supports the critical role of infection in the pathogenesis of severe cerebral hemorrhage. These results suggest that systemic and central nervous system infections may be associated with increased IVH severity, possibly through inflammatory responses and hemodynamic instability. Therefore, early diagnosis, close monitoring, and appropriate management of neonatal sepsis and meningitis are essential not only for reducing the risk of severe IVH but also for preventing infection-related cerebral injury and improving neurodevelopmental outcomes.

## Figures and Tables

**Table 1:** Demographic, clinical, and laboratory characteristics of neonates diagnosed with intraventricular hemorrhage (IVH).

Variable	Statistics
**Gender** **, ** ***n*** ** (%)**	
Female	24 (39.3)
Male	37 (60.7)
**Gestational age, weeks, median (IQR)**	30.0 (27.0–31.0)
**Gestational age category, ** ***n*** ** (%)**	
<28 weeks	18 (29.5)
≥28 to <32 weeks	32 (52.5)
≥32 to <36 weeks	3 (4.9)
≥36 weeks	8 (13.1)
**Birth weight, g, median (IQR)**	1405 (970–1888)
**Birth weight category, ** ***n*** ** (%)**	
<1000 g	16 (26.2)
≥1000 to <1500 g	18 (29.5)
≥1500 to <2500 g	18 (29.5)
≥2500 g	9 (14.8)
**Mode of delivery, ** ***n*** ** (%)**	
Cesarean section	51 (83.6)
Vaginal delivery	10 (16.4)
**Time from birth to diagnosis of IVH, median (IQR), days**	9 (3–25)
**Imaging modality, ** ***n*** ** (%)**	
Ultrasonography (USG)	52 (85.2)
Magnetic Resonance Imaging (MRI)	5 (8.2)
Computed Tomography (CT)	4 (6.6)
**IVH Grade, median (IQR)**	3 (3–4)
**Sepsis, ** ***n*** ** (%)**	37 (60.7)
**Blood culture result, ** ***n*** ** (%)**	
Gram-negative bacteria	18 (29.5)
Gram-positive bacteria	14 (23.0)
Fungal	3 (4.9)
No growth	26 (42.6)
**Mortality, ** ***n*** ** (%)**	13 (21.3)
**Hemoglobin, mean ± SD (g/dL)**	14.9 ± 4.0
**INR, median (IQR)**	1.4 (1.1–1.9)
**Platelet count, mean ± SD (×10^3^/µL)**	195.1 ± 111.2
**C-reactive protein, median (IQR), mg/L**	14.5 (5.0–50.0)
**CSF protein, median (IQR), mg/dL**	1442 (829–2977)
**CSF glucose, median (IQR), mg/dL**	32.0 (13.5–53.5)
**CSF WBC, median (IQR), cells/µL**	0 (0–40)
**CSF RBC, median (IQR), cells/µL**	0 (0–1000)
**CSF culture, ** ***n*** ** (%)**	
Positive	6 (9.8)
Negative	55 (90.2)
**Organism isolated from CSF, ** ***n*** ** (%)**	
Gram-negative bacteria	4 (6.6)
Gram-positive bacteria	1 (1.6)
Fungal	1 (1.6)
No growth	55 (90.2)

Abbreviations: CSF, cerebrospinal fluid; IVH, intraventricular hemorrhage; MRI, magnetic resonance imaging; CT, computed tomography; USG, ultrasonography; RBC, red blood cell; WBC, white blood cell; INR, international normalized ratio; SD, standard deviation; IQR, interquartile range.

**Table 2:** Comparison of clinical, laboratory, and infection-related parameters between low-grade and high-grade intraventricular hemorrhage (IVH) groups.

Variable		Low-Grade IVH (*n* = 12)	High-Grade IVH (*n* = 49)	*p* Value
**Sex, ** ***n*** ** (%)**				
	Female	8 (66.7)	16 (32.7)	***p*** ** = 0.047 †**
	Male	4 (33.3)	33 (67.3)	
**Gestational age, ** ***n*** ** (%)**				
	<28 weeks	1 (8.3)	17 (34.7)	*p* = 0.063 ‡
	≥28 to <32 weeks	8 (66.7)	24 (49.0)	
	≥32 to <36 weeks	2 (16.7)	1 (2.0)	
	≥36 weeks	1 (8.3)	7 (14.3)	
	Gestational age, mean ± SD (weeks)	31.2 ± 3.5	29.7 ± 4.1	*p* = 0.074 §
	Birth weight, mean ± SD, g	1708.8 ± 734.9	1567.0 ± 832.6	*p* = 0.355 §
**Birth weight, median (IQR), g**		1685 (1212.5–1960)	1360 (890–1850)	*p* = 0.350 §
**Birth weight category, ** ***n*** ** (%)**				
	<1000 g	1 (8.3)	15 (30.6)	*p *= 0.443 ‡
	≥1000 to <1500 g	2 (16.7)	14 (28.6)	
	≥1500 to <2500 g	5 (41.7)	13 (26.5)	
	≥2500 g	2 (16.7)	7 (14.3)	
**Time from birth to diagnosis, median (IQR), days**		11.0 (4.0–19.5)	9.0 (3.0–25.0)	*p* = 0.669 §
**Sepsis, ** ***n*** ** (%)**				
	Present	4 (33.3)	33 (67.3)	***p *** **= 0.047 †**
**Blood culture result, ** ***n*** ** (%)**				
	Gram-negative bacteria	3 (25.0)	15 (30.6)	*p* = 0.691 ‡
	Gram-positive bacteria	4 (33.3)	10 (20.4)	
	Fungal	1 (8.3)	2 (4.1)	
	No growth	4 (33.3)	22 (44.9)	
**Mode of delivery, ** ***n*** ** (%)**				
	Cesarean section	9 (75.0)	42 (85.7)	*p* = 0.397 †
	Vaginal delivery	3 (25.0)	7 (14.3)	
**Mortality, ** ***n*** ** (%)**				
	Death	3 (25.0)	10 (20.4)	*p* = 0.707 †
**Hemoglobin, mean ± SD (g/dL)**		15.4 ± 5.3	14.7 ± 3.7	*p* = 0.617 ¶
**INR, median (IQR)**		1.40 (1.15–1.55)	1.40 (1.10–2.00)	*p* = 0.533 §
**Platelet count, mean ± SD (×10^3^/µL)**		186.3 ± 101.8	197.3 ± 114.3	*p* = 0.762 ¶
**C-reactive protein, median (IQR), mg/L**		7.75 (4.00–24.85)	16.90 (5.00–50.00)	*p* = 0.399 §
**CSF protein, median (IQR), mg/dL**		1500 (46–1907)	1434 (833–2977)	*p* = 0.608 §
**CSF glucose, median (IQR), mg/dL**		80 (49–104)	24 (11–41)	***p *** **= 0.002 §**
**CSF WBC, median (IQR), cells/µL**		0 (0–0)	0 (0–40)	*p* = 0.748 §
**CSF RBC, median (IQR), cells/µL**		0 (0–1500)	0 (0–750)	*p* = 0.665 §
**CSF culture, ** ***n*** ** (%)**				
	Negative	12 (100.0)	43 (87.8)	*p* = 0.588 †
	Positive	0 (0.0)	6 (12.2)	
**Organism isolated from CSF, ** ***n*** ** (%)**				
	Gram-negative bacteria	0 (0.0)	4 (8.2)	*p* = 0.653 ‡
	Gram-positive bacteria	0 (0.0)	1 (2.0)	
	Fungal	0 (0.0)	1 (2.0)	
	No growth	12 (100.0)	43 (87.8)	
**Gestational age, ** ***n*** ** (%)**				
	<32 weeks	9 (75.0)	41 (83.7)	*p* = 0.676 †
	≥32 weeks	3 (25.0)	8 (16.3)	

Abbreviations: IVH, intraventricular hemorrhage; CSF, cerebrospinal fluid; IQR, interquartile range. † Chi-square test, ‡ Fisher’s exact test, § Mann–Whitney U test, ¶ Student’s *t* test.

**Table 3:** Logistic regression analysis of factors associated with high-grade intraventricular hemorrhage (IVH).

Variable	Odds Ratio (OR)	95% Confidence Interval (CI)	*p*-Value
**Sex (Male)**	4.125	1.080–15.762	0.038
**Gestational age (Ref: <28 weeks)**			
≥28 to <32 weeks	0.176	0.020–1.545	0.117
≥32 to <36 weeks	0.029	0.001–0.676	0.027
≥36 weeks	0.412	0.022–7.545	0.550
**Birth weight**	1.000	0.999–1.001	0.585
**Birth weight category (Ref: <1000 g)**			
≥1000 to <1500 g	0.233	0.023–2.349	0.217
≥1500 to <2500 g	0.173	0.018–1.681	0.131
≥2500 g	0.233	0.018–3.026	0.266
**Sepsis**	4.125	1.080–15.762	0.038
**CSF protein**	1.000	1.000–1.000	0.981
**CSF glucose**	0.983	0.966–1.000	0.054
**CSF WBC**	1.004	0.993–1.014	0.519
**CSF RBC**	1.000	1.000–1.000	0.105

Abbreviations: IVH, intraventricular hemorrhage; CSF, cerebrospinal fluid; WBC, white blood cell; RBC, red blood cell; OR, odds ratio; CI, confidence interval.

## Data Availability

The data supporting the findings of this study are available from the corresponding author upon reasonable request. The data are not publicly available due to privacy and ethical restrictions.
